# The effectiveness of high intensity intermittent training on metabolic, reproductive and mental health in women with polycystic ovary syndrome: study protocol for the iHIT- randomised controlled trial

**DOI:** 10.1186/s13063-019-3313-8

**Published:** 2019-04-16

**Authors:** Danielle Hiam, Rhiannon Patten, Melanie Gibson-Helm, Alba Moreno-Asso, Luke McIlvenna, Itamar Levinger, Cheryce Harrison, Lisa J Moran, Anju Joham, Alex Parker, Soulmaz Shorakae, David Simar, Nigel Stepto

**Affiliations:** 1 Institute for Health and Sport, Victoria University, PO Box 14428, Melbourne, Victoria 8001 Australia; 2 Monash Centre for Health Research and Implementation, School of Public Health and Preventive Medicine, Monash University, Melbourne, Australia; 3 Australian Institute for Musculoskeletal Science (AIMSS), Department of Medicine-Western Health, Melbourne Medical School, The University of Melbourne, Melbourne, VIC Australia; 4 Mechanisms of Disease and Translational Research, School of Medical Sciences, UNSW Sydney, Sydney, Australia

**Keywords:** Polycystic ovary syndrome, High-intensity interval training, Cardiorespiratory fitness, Cardiometabolic health, Insulin, Mental health, Overweight, Exercise, Exercise therapy

## Abstract

**Background:**

Polycystic ovary syndrome (PCOS) is a reproductive-metabolic condition. Insulin resistance is a hallmark of PCOS and is related to increased hyperandrogenism that drives inherent metabolic, reproductive and psychological features of the syndrome. Insulin resistance in women with PCOS is managed by weight loss, lifestyle interventions (i.e. exercise, diet) and insulin-sensitising medications. This manuscript describes the protocol of our study evaluating the effectiveness of high intensity intermittent training (HIIT) or moderate intensity exercise on cardiometabolic, reproductive and mental health in overweight women with PCOS.

**Methods/design:**

We will employ a three arm, parallel-group, randomised controlled trial recruiting 60 women diagnosed with PCOS, aged between 18 and 45 years and with a body mass index (BMI) greater than 25 kg/m^2^. Following screening and baseline testing, women will be randomised by simple randomisation procedure using computer generated sequence allocation to undergo one of two 12-week supervised interventions: either HIIT or moderate intensity exercise (standard supervised exercise), or to standard care [Con] (unsupervised lifestyle advice) at a 1:1:1 allocation ratio. The primary outcome for this trial is to measure the improvements in metabolic health; specifically changes in insulin sensitivity in response to different exercise intensities. Baseline and post-intervention testing include anthropometric measurements, cardiorespiratory fitness testing, reproductive hormone profiles (anti-müllerian hormone and steroid profiles), metabolic health, health-related quality of life and mental health questionnaires and objective and subjective lifestyle monitoring. Reporting of the study will follow the CONSORT statement.

**Discussion:**

This trial aims to demonstrate the comparative efficacy and maintenance of different exercise intensities to advance the understanding of PCOS management and provide insight into the optimal exercise intensity for improved cardiometabolic outcomes. Secondary outcomes will include the impact of different exercise protocols on reproductive hormone profiles, mental health and health-related quality of life.

**Trial registration:**

Australian New Zealand Clinical Trials Registry, ACTRN12615000242527. Registered on 17 March 2015.

**Electronic supplementary material:**

The online version of this article (10.1186/s13063-019-3313-8) contains supplementary material, which is available to authorized users.

## Background

Polycystic ovary syndrome (PCOS) is a major public health concern affecting 6–10% of reproductive-aged women worldwide [1]. Insulin resistance is strongly implicated in the aetiology of PCOS and is associated with the reproductive and metabolic consequences of the syndrome [2–7]. Insulin resistance in PCOS is inherent and has been demonstrated to be independent of body mass index (BMI), yet can be exacerbated by obesity [2, 8], although the mechanisms remain unclear. Lifestyle interventions (diet, exercise and/or behavioural) and weight management are the first line therapy [9] for women with PCOS as they improve the clinical symptoms by increasing insulin sensitivity in these women [4, 10].

Moderate intensity aerobic exercise improves metabolic and reproductive features, body composition and psychological well-being in overweight women with PCOS [4, 11, 12]. However, even after exercise interventions a higher level of insulin resistance prevails in women with PCOS compared to women without PCOS, indicating further research is warranted [4]. High intensity interval training (HIIT) is a popular fitness program that encompasses short bouts of high intensity exercise interspersed with active recovery [13]. HIIT has been shown to be acceptable and safe and may assist in addressing barriers reported in women of reproductive age, including time limitations and competing commitments and PCOS-specific barriers such as low confidence and physical limitations [14, 15]. HIIT is enjoyable [4, 16–18] and has been found to have a superior effect on insulin resistance, cardiovascular risk factors and all-cause mortality amongst other predominantly male and older clinical populations [19–22]. Despite this, there is limited comprehensive research on the efficacy of different exercise intensities and optimal exercise intensity for improved cardiometabolic outcomes, reproductive hormone profiles, mental health and health-related quality of life following exercise in PCOS.

Compared to recommended lower-intensity regimes, more positive metabolic health outcomes are now being reported for HIIT, including improved glycaemic control and cardio-respiratory fitness in clinical populations and amongst women with PCOS [17, 18, 22–24]. Only one randomised controlled trial (RCT) has been conducted in women with PCOS to assess the benefits of HIIT, in which they compared a resistance-training program and a control group [18]. After 10 weeks, they found improvements in insulin sensitivity and high-density lipoprotein cholesterol and a decrease in fat percentage [18]. A cross-sectional study conducted by Greenwood et al. [23] was the first to examine the effect of exercise of varying intensities amongst women with PCOS. The results of their study supported superior health benefits of vigorous exercise, including a lower BMI and Homeostatic Model Assessment of Insulin Resistance (HOMA-IR) and higher levels of high-density lipoprotein and sex hormone binding globulin compared with moderate exercise in women with PCOS, independent of age, BMI and total exercise volume [23]. Specifically, it was found that 60 min of vigorous activity per week was associated with a 22% reduction in the odds ratio of metabolic syndrome (1.11; 95% confidence interval 1.04, 1.18) [23]. Although these studies provide positive results in favour of high intensity exercise, more objective data and further investigation are required to find the optimal intensity in promoting the greatest health benefits for women with PCOS. Therefore, the aim of this protocol paper is to detail an exercise intervention that explores the clinical effectiveness and efficacy of HIIT compared to moderate intensity exercise or standard care (unsupervised lifestyle intervention) on metabolic (body composition and insulin sensitivity), reproductive (anti-müllerian hormone [AMH] and steroid profiles) and mental health (depression and health-related quality of life) in women with PCOS.

## Methods/design

### Hypothesis

Based on previous literature, we hypothesise that HIIT will result in greater health outcomes such as greater insulin sensitivity, increased lean muscle mass, decreased androgens, increased sex hormone binding globulin and improved mental health, and health-related quality of life compared to moderate intensity exercise or standard care.

### Design

**Fig. 1:**
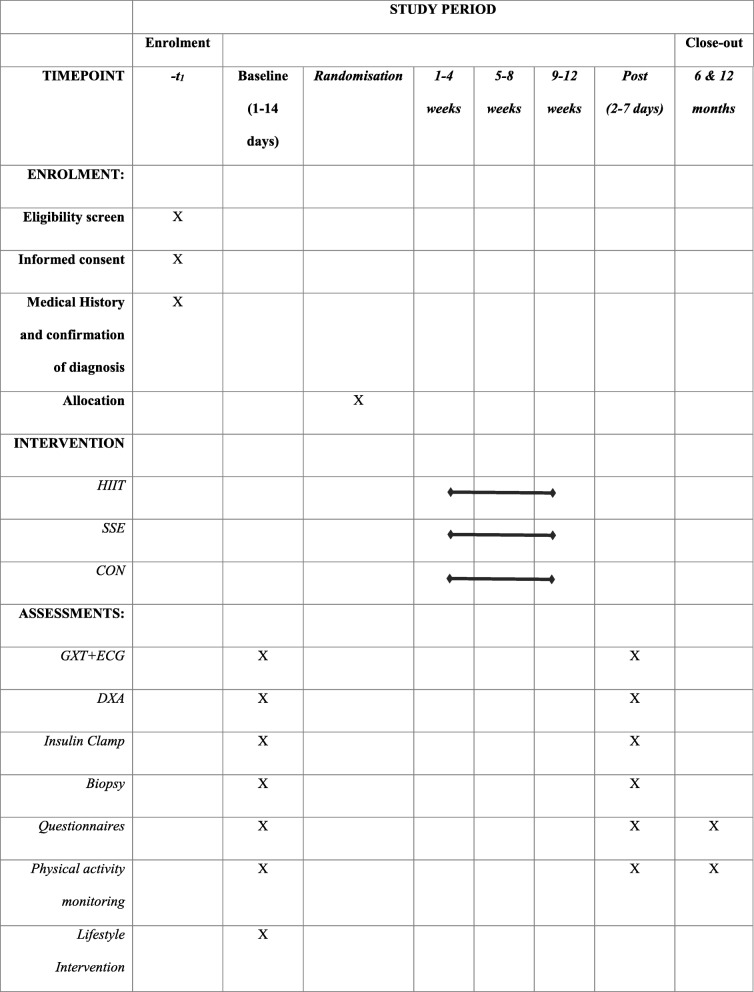
SPIRIT flow diagram of randomised control trial. *GXT* graded exercise test, *ECG* electrocardiogram, *Insulin clamp* hyperinsulinemic-euglycemic clamp, *DXA* dual energy x-ray absorptiometry, *HIIT* high intensity intermittent training, *SSE* supervised standard exercise, *Con* control

We will employ a three-arm, parallel-group, RCT (Fig. 1) and recruit 60 women diagnosed with PCOS from the community and health clinics that are located in Melbourne, Australia, via advertisement flyers in local media, information boards in clinics and PCOS websites. They will be randomised to undergo one of three 12-week supervised interventions: either HIIT, standard supervised exercise (SSE), or standard care [Con] (unsupervised lifestyle advice) with a 6-month and 12-month post-intervention follow-up for selected endpoints.

### Inclusion criteria

Women aged 18–45 years with a BMI greater than 25 kg.m^2^ and diagnosed with PCOS using the Rotterdam criteria [25] will be recruited for this study. Their medical practitioner will have previously diagnosed PCOS and the research endocrinologist will confirm this prior to participation in the study. Diagnosis of PCOS by the Rotterdam criteria requires confirmation of two of the following: (i) oligo- or anovulation; (ii) clinical (hirsutism and acne) and/or biochemical hyperandrogenism; (iii) polycystic ovaries on ultrasound and exclusion of other causes of hyperandrogenism [25]. Features of PCOS will be recorded to allow phenotyping as recommended by the National Institutes of Health [26].

### Exclusion criteria

Exclusion criteria will be other causes of menstrual disturbance and hyperandrogenism, known cardiovascular (cardiac arrhythmias), uncontrolled hypertension (resting systolic blood pressure > 160 mmHg and/or diastolic blood pressure > 105 mmHg), bleeding disorders, skin or anaesthetic allergies, musculoskeletal injuries that may be aggravated by the exercise protocol, pregnancy, type 1 or 2 diabetes or taking anti-hypertensive, insulin sensitising, anti-obesity or hormonal contraceptive medications.

### Screening

Prospective participants will be screened to check eligibility and to assess potential risks of adverse events during exercise. If identified as having multiple risk factors (family history [first-degree] of cardiometabolic risk factors, chronic conditions [that do not exclude from participation] but could interfere with testing or exercise, or have a BMI > 40 kg.m^2^), exercise clearance will be required by their general practitioner. A member of the research team will obtain written informed consent from all participants prior to participation in the study.

### Data monitoring

Details of procedures for data management have been reviewed and approved by the Victoria University Human Research Ethics Committee (reference HRE15–298). All electronic data will be stored on password-protected computers. Hard copies of any data will be kept in locked filing cabinets in a secure office. All questionnaire data will be de-identified.

#### Harms

All adverse events associated with the study will be recorded and be reported to the Victoria University Human Research Ethics Committee.

#### Auditing

No formal auditing process is proposed for the current trial. The principal investigator, Professor Nigel Stepto, has extensive experience as a lead research investigator in numerous human clinical trials and is responsible for the integrity of the current trial and [3, 12, 27].

### Ethics

The study has been approved by the Victoria University Human Research Ethics Committee (reference HRE15–298) and is registered with the Australian New Zealand Clinical Trials Registry (ACTRN12615000242527). Any modifications or changes to the protocol will be provided in writing to the Victoria University Human Research Ethics Committee for approval. The study protocol was designed in accordance with the SPIRIT checklist (Additional file 1). Reporting of the study will follow the CONSORT statement [28].

### Baseline assessment

Baseline assessment will involve three sessions. Two sessions will be required to complete a symptom-limited graded exercise protocol (the first session being a familiarisation of the test). In the 7 days preceding session three, participants will wear an Actigraph™ accelerometer to establish levels of habitual physical activity. The third baseline session will involve a muscle biopsy from the vastus lateralis, an adipose tissue biopsy from the subcutaneous abdominal tissue and a hyperinsulinemic-euglycemic clamp. Participants will also undergo a dual x-ray absorptiometry scan (DXA; GE Lunar iDXA) and be asked to complete validated questionnaires to assess physical activity (International Physical Activity Questionnaire [IPAQ]) [29], quality of life (SF-36 & PCOSQ) [30, 31], depression, anxiety and stress (Depression Anxiety Stress Scale [DASS-21]) [32] and a 3-day food diary.

### Randomisation and blinding

Following baseline testing, participants will be randomised to one of three 12-week interventions; HIIT, SSE or Con (details of the three treatment arms in Table 1). An independent biostatistician will randomise subjects by a simple randomisation procedure using computerised sequence generation with an allocation ratio of 1:1:1. Two tables based on BMI (< 35 kg.m^2^ or > 35 kg.m^2^) will be created. As exercise interventions cannot be blinded from participants and staff (accredited exercise physiologists) implementing the intervention, only staff undertaking sample analysis and endpoint data processing will be blinded to group allocation.

**Table 1:** Details of the three treatment arms

Intervention	Details
Con	Standard care consisting of basic exercise advice (150 min/week) with no supervised or structured exercise. We do not anticipate the control group will engage in a significant amount of exercise compared to their baseline physical activity levels without the addition of a structured exercise program [11]
SSE group	Minimum physical activity recommendations (150 min per week) [40], in three supervised 50-min sessions/week of continuous moderate intensity exercise sessions of cycling at 50–60% HRR (~ 3.5 METs)
HIIT group	Minimum vigorous physical activity recommendations (~ 75 min per week) [40], in three supervised sessions/week of HIIT exercise (cycling/running). Based on existing literature, pilot data [43] and patient consultation, we will use a practical weekly training program encompassing two successful HIIT protocols [16, 17, 55]: two sessions/week of short constant load cycling of 12 × 1 min at > 85% HRR (> 9 METs; [1 min HIIT]) with 1 min active recovery; one session/week of cycling 6–8 × 4 min at > 85% HRR (> 9 METS; [4 min HIIT]) with 2 min active recovery

*Con* control- standard care, *SSE* supervised standard exercise, *HIIT* high-intensity intermittent training, *min* minutes, *MET* metabolic equivalent task, *HRR* heart rate reserve

### Intervention

Every participant will receive a menstrual diary to monitor menstrual cycles. As recommended by the Australian evidence-based PCOS guidelines and International evidence-based PCOS guidelines, all women will receive evidence-based behaviour change coaching [9, 10] based on social cognitive theory and the behaviour change wheel [33]. This will involve a 2-h session including goal setting and goal striving, education regarding the importance of physical activity and healthy eating and education on effectively using media and resources for healthy eating and diet [34].

### Exercise interventions

Exercise will be conducted on stationary bikes in an individual setting. Exercise intensities will be prescribed and monitored using heart rates (percentage of heart rate reserve [HRR]), as described in Table 1. Sessions will be conducted at Victoria University fitness centres/exercise clinics under the supervision of accredited exercise physiologists [4, 12, 35–39]. Adherence and compliance will be determined from supervised exercise session attendance and completion of prescribed exercise (duration and intensity [percentage HRR]), respectively. Data from participants with less than 75% adherence will be included in the intention to treat analysis only. Any musculoskeletal injuries or changes in health status will be recorded via a provided web-based diary.

#### Volume matching and training progression

The SSE and HIIT intervention arms will be matched for training volume (metabolic equivalent task (MET).min/week) and progressed weekly by manipulating session duration and intensity. Both SSE and HIIT will progress from 312 MET.min/week in week 1 to 530 MET.min/week in weeks 6–12, meeting exercise guidelines [40]. Exercise sessions will include warm-up and cool down protocols, and will be adjusted weekly to individual capabilities and training adaptations [4, 41]. See Table 1 for details.

#### Preventing and managing injury

Absolute risk of cardiovascular disease in this young female population is low. However, participants will be screened with appropriate clinical monitoring during all exercise testing (ECG) and training sessions (heart rate and blood pressure monitoring) with individualised training prescription and progression reducing the risks of injuries and adverse events [4, 12, 35, 36].

### Post-intervention assessment

Within 4 days after the final exercise session (HIIT or SSE) or following 12 weeks (Con) the hyperinsulinemic-euglycemic clamp, questionnaires (SF-36, PCOSQ, IPAQ and DASS-21), muscle and fat biopsies, body composition measures (DXA, weight, waist and hip circumferences) and graded exercise tests will be repeated. Women will be asked to abstain from training or physical exercise during this period. An accelerometer will be given (or mailed if in the control group) 7 days before post-intervention testing to assess exercise in this period. They will again be asked to complete a 3-day food dairy.

#### Follow-up

Six and 12 months after completion of the 12-week intervention phase, women will return to study centres for re-assessment of selected endpoints (mental health, health-related quality of life and physical activity questionnaires) to determine whether participants continued healthy lifestyle behaviours after the conclusion of the intervention.

### Outcome measurements

Outcome measures will be taken pre-intervention and post-intervention (Fig. 1). The primary outcome is the change in insulin sensitivity (measured by the steady state glucose infusion rate determined during the hyperinsulinemic-euglycemic clamp) between the three interventions. Secondary outcomes include reproductive health via hormone profiles (AMH and steroid profiles), mental health and health-related quality of life, body composition, physical activity behaviours and cardio-respiratory fitness.

## Data collection and analysis

### Anthropometric assessment

Following an overnight fast, participants will be weighed lightly clothed and without shoes (HW-PW200, associated scales services). Height will be taken without shoes using a calibrated stadiometer (Proscale Inductive Series I, Accurate Technology Inc.) and BMI calculated [weight (kilograms)/height squared (squared metres)]. Waist and hip circumference measurements will be taken [42] and the waist to hip ratio calculated. Fat mass, abdominal fat mass and fat free mass will be assessed using DXA (iDXA GE Lunar Prodigy scanner) and analysed by a qualified operator.

### Fitness parameters

Cardiorespiratory fitness via peak oxygen uptake (VO_2peak_) will be assessed using a symptom-limited graded exercise protocol on a cycle-ergometer [43]. The test will start after a 5-min period at rest. The protocol will consist of three 3-min stages at an intensity of 25 watts (W), 50 W and 75 W, respectively, and then an increase by 25 W every minute. The test will be terminated objectively when:

- Participant can no longer sustain a pedal rate greater than 60 rpm
- There is no longer a change in VO_2_ with increasing work rate
- Respired expiratory rate reaches 1.1 or greater
- Appearance of clinical signs or symptoms of metabolic or cardiorespiratory abnormalities
- Patient wishes to stop

Expired respiratory gases will be collected by the COSMED cardio pulmonary exercise test system breath-by-breath connected to automated gas analysers. The system will be calibrated before conducting each test using Hans Rudolph syringe and gases of known O_2_ and CO_2_ content (BOC gas). As a precautionary measure, 12-lead ECG will be monitored to minimise adverse events from undiagnosed cardiac arrhythmias during this testing.

### Physical activity and diet

Physical activity and exercise before baseline testing and before post-intervention testing will be monitored by a triaxial accelerometer (Actigraph™) for 7 continuous days. Average daily time spent in moderate to vigorous activity and METs will be calculated by the Freedson VM3 (2011) algorithms in Actlife software [44]. During the 12-week intervention habitual physical activity will be objectively assessed continuously using physical activity monitors (FitBit Flex™) and a smart phone application. Dietary habits will be assessed by a consecutive 3-day food diary before baseline and post-intervention testing. Food diaries will be analysed by FoodWorks® (Xyris) for the major food groups (grains, fruit, vegetables, protein and dairy), total energy and macronutrients.

### Self-reported measures

Participants will monitor their menstrual cycles using a menstrual diary, which will be used to assess menstrual cyclicity throughout the study. Self-reported physical activity questionnaires (IPAQ) [29] will be used to assess physical activity behaviour pre- and post-intervention. Mental health and health-related quality of life measures will be completed pre- and post-intervention to identify any changes in mental health and wellbeing after an exercise intervention and to determine the relationship with other outcome measures (DASS, SF-36 and PCOSQ) [30, 32, 45].

### Hyperinsulinemic-euglycemic clamp

Participants will undergo a hyperinsulinemic-euglycemic clamp to measure insulin sensitivity [3, 46, 47]. Human insulin (NovoNordisk ActRapid) will be infused at a constant rate (40 mU/min/m^2^) while a variable rate glucose solution is infused to meet the target of 5 mmol.L^− 1^ blood glucose in the last 30 min of the clamp. During the clamp, one hand will be warmed to improve blood flow for blood sampling. Blood samples will be taken every 5 min to monitor circulating blood glucose and an additional blood sample will be taken every 30 min during the clamp for insulin analysis. To reduce the risk of low potassium levels (hypokalaemia), participants will be given a single dose (600 mg) of slow-release potassium before the commencement of the insulin clamp.

### Pathology analysis

Glucose will be measured using an automated analyser (YSI 2300 STAT Plus). Insulin and N-terminal pro-peptide of type I and III collagen (collagen synthesis biomarkers) concentrations will be determined by radioimmunoassay according to manufacturer instructions (HI-14 K, EMD Millipore, UniQ PIIINP RIA #68570, UniQ PINP RIA #67034, Orion Diagnostica). Standard clinical pathology testing, including lipid profiles, haemoglobin A1c (HbA1c) and AMH, will be performed by a health pathology service. Serum steroid profiles, including testosterone, dihydrotestosterone, 3α and 3β androstanediols, estradiol, estrone, dehydroepiandrosterone (DHEA), androstenedione and progesterone, will be determined by liquid chromatography–mass spectrometry. Enzyme-linked immunosorbent assay (ELISA) will be used to measure transforming growth factor beta 1/3 (TGFβ1/3). To minimize variability, samples will be stored at (− 80 °C) and batch analysed by a single laboratory.

### Tissue biopsies

Vastus lateralis muscle and peri-umbilical fat biopsies will be carried out by a medical practitioner under local anaesthesia. After a local anaesthetic (1% xylocaine) is injected under the skin, a small incision is made to access the thigh muscle or peri-umbilical fat tissue and a small amount of each tissue is extracted from a consistent depth using a Bergstrom biopsy needle with suction [46]. Tissue samples will be used for the determination of DNA methylation profiles and protein levels of key tissue fibrosis molecules, insulin signalling proteins and TGFβ ligands before and after the 12-week intervention by western blotting.

### Western blotting

Protein levels of key tissue fibrosis molecules, insulin signalling proteins and TGFβ ligands will be measured by western blotting. Protein extraction and western blotting will be performed as previously described [27].

### Global methylation of peripheral blood mononuclear cell populations

Detailed methodology of peripheral blood mononuclear cell (PBMC) isolation and quantification has previously been described [48]. Briefly PBMCs will be isolated and collected by centrifugation by Ficoll gradient, before being stained for flow cytometry. A specific gating strategy to analyse T-helper cells, T cytotoxic cells, B cells and monocytes will be used to analyse the 5-methylcytosine quantity in each immune cell population.

### Statistical methods and determination of sample size

Primary analyses will be undertaken on an intention-to-treat basis, including all participants as randomised, regardless of treatment actually received. The HIIT group will be compared with the SSE and Con groups using a planned contrast of change from baseline to the week 12 endpoint on the basis of insulin sensitivity scores using a mixed-model repeated measures analysis. Stratification variables will be evaluated and retained in analyses where they are measured as significant or quasi-significant. Transformation of scores, including categorisation, may be undertaken to meet distributional assumptions and accommodate outliers. Comparisons of changes in insulin sensitivity scores from baseline to other time points will be undertaken as secondary analyses. Data will be analysed by SPSS and significance will be accepted when *p* < 0.05. Where required *p* values from the statistical analysis will be adjusted for multiple comparisons using the false discovery rate [49] and statistical significance will be accepted when false discovery rate q < 0.1.

Using the primary outcome of change in insulin sensitivity (as measured by the glucose infusion rate) of 27 mg/min/m^2^ (effect size (Cohens d) = 0.25) in response to training [4], we require a total of 42 participants or *n* = 14 per group to achieve a power of 85% (α = 0.05). With an approximate attrition rate of 30% based on previous research, we will recruit 60 participants [4, 12].

## Discussion

PCOS is under-recognised by health professionals and leaves women on track for a plethora of chronic conditions ranging from anxiety and depression to diabetes, subfertility, cardiovascular disease and stroke [1, 50–52]. Despite affecting around one million Australian women and costing over $800 million nationally each year [10, 53, 54], there are no optimal treatments.

Overall, HIIT in PCOS promises greater metabolic benefits with demonstrated acceptability and safety [23]. It has the potential to address general and PCOS-specific barriers (low confidence and physical limitations) to standard exercise participation [14, 15]. However, evidence from prospective studies comparing volume-matched HIIT and standard moderate exercise for efficacy and enjoyment is lacking in women generally and more importantly in women at high risk of metabolic disease (i.e. PCOS).

Here, we describe the protocol of a study evaluating the effectiveness and mechanistic impacts of a practical allied health supervised 12-week HIIT or moderate intensity exercise program on cardiometabolic, reproductive and mental health in overweight women with PCOS. This trial aims to demonstrate comparative efficacy of different exercise protocols to inform vital large-scale clinical trials and ultimately best clinical practice in treatment of PCOS. It will advance the understanding of PCOS management by providing insights into the best exercise intensities to improve insulin sensitivity. Further helping to address the limitations highlighted by the recently released international guidelines in PCOS lifestyle management [9]. Finally, we will explore the impact of different exercise protocols on reproductive health (anti-müllerian hormone and steroid profiles), mental health and health-related quality of life and molecular mechanisms that affect insulin resistance. The impact of this work is likely to be significant due to the unprecedented public health challenge of PCOS in young Australian women for which we currently have no optimal treatment.

## Additional file

Additional file 1: SPIRIT Checklist. (DOC 115 kb)

## Abbreviations

AMH: Anti-müllerian hormone
BMI: Body mass index
Con: Standard care
DASS: Depression Anxiety Stress Scale
DXA: Dual energy x-ray absorptiometry
ECG: Electrocardiogram
GXT: Graded exercise test
HIIT: High intensity intermittent training
HRR: Heart rate reserve
Insulin clamp: Hyperinsulinemic-euglycemic clamp
IPAQ: International Physical Activity Questionnaire
METs: Metabolic equivalent task
PBMC: Peripheral blood mononuclear cell
PCOS: Polycystic ovary syndrome
RCT: Randomised controlled trial
SSE: Standard supervised exercise (moderate intensity exercise)
TGFβ1/3: Transforming growth factor beta 1/3
